# Applied research won’t flourish without basic science

**DOI:** 10.7554/eLife.102368

**Published:** 2024-09-10

**Authors:** Jon R Lorsch, Lawrence A Tabak, Monica M Bertagnolli

**Affiliations:** 1 National Institute of General Medical Sciences, National Institutes of Health Bethesda United States; 2 National Institutes of Health Bethesda United States

**Keywords:** Point of View, National Institutes of Health, science funding, basic research, early-career researchers, None

## Abstract

Three senior figures at the US National Institutes of Health explain why the agency remains committed to supporting basic science and research.

It often seems as if there is a pendulum that swings back and forth in science policy circles, sometimes favoring curiosity-driven basic science, and other times championing outcomes-based applied research. At the moment, applied research seems to be favored, with much attention focused on how the research enterprise can most effectively translate existing scientific information into solutions to real-world problems, including new diagnostics and therapies for diseases. Examples of this in the United States include the Advanced Research Projects Agency for Health (ARPA-H), which was set up in 2022, and CARE for Health, a primary care-based clinical research network that was established by the National Institutes of Health (NIH) earlier this year.

While applied research appears to be in the ascendancy at the moment, it is important to remember that without an equal and ongoing commitment to basic science, there would soon be nothing to translate into applications. As Vannevar Bush wrote in Science, the Endless Frontier: ‘Basic research leads to new knowledge. It provides scientific capital. It creates the fund from which the practical applications of knowledge must be drawn. New products and new processes do not appear full-grown. They are founded on new principles and new conceptions, which in turn are painstakingly developed by research in the purest realms of science’.

NIH remains deeply committed to sustaining basic research, and it currently spends about 51% of its annual budget on such research. All of the agency’s institutes, centers and offices fund or otherwise support basic science studies (Collins et al., 2016), and the success of this strategy can be seen in many different ways. For example, 171 scientists supported by the NIH have received a Nobel Prize, including 94 who were supported by the National Institute of General Medical Sciences (NIGMS), which primarily funds basic research.

We cannot predict in advance what lines of inquiry will produce information that will eventually be important in enabling an application.

Historical examples of basic science work that led eventually to applied breakthroughs include the discovery of the anticancer drug cisplatin by Barnett Rosenberg of Michigan State University while he was studying the effects of electric fields on bacterial cell division (Petsko, 2002), work on CRISPR – a system for protecting bacteria against viruses – which has now been harnessed into gene editing and control technologies that are already being used in clinical applications to treat sickle cell disease and beta-thalassemia (Ishino et al., 2018), and the development and production of the mRNA vaccines for COVID-19, which relied at every stage on basic science, including research into protein-protein interactions and conformational changes (Casadevall, 2021; Wrapp et al., 2020).

## How best to support basic science

When considering the best approaches for supporting basic science, it is important to recognize several key principles. First, we cannot predict in advance what lines of inquiry will produce information that will eventually be important in enabling an application. Second, it can be many years before a strand of fundamental knowledge – which might at first seem of interest only to a small number of academic experts – becomes critical for developing a new therapeutic agent or other application. Finally, it is impossible to predict where important discoveries or insights will take place or who will be involved in making them. Demonstrating these principles, it has been shown that the development of a new drug can rely on the work of thousands of scientists at thousands of organizations over many decades (Williams et al., 2015).

Because of these principles, it is essential that NIH supports a broad portfolio of basic research – broad across research questions and systems, institutions, and investigators. We must encourage researchers to delve into unexplored areas of biology, including working on organisms that are not ‘the usual suspects’ but could be hiding unknown pathways that might eventually be as important in applied research as CRISPR is today (Sánchez Alvarado, 2018). Who knows what secrets the banded panther worm, African spiny mouse, axolotl, or organisms yet to be found contain? We must also continue to deepen and refine our understanding of fundamental biological processes because these details frequently hold the keys to major advances in applied research. For example, recent progress in immunotherapies for cancer, autoimmune diseases, and chronic infections rely on detailed mechanistic understanding of cell signaling processes and immunological control pathways (Waldman et al., 2020).

It is also crucial that NIH maintains its longstanding commitment to training future generations of basic biomedical researchers.

To advance basic research on these and other fronts, we must harness the virtuous cycle between scientific research and technology development, and create new instruments and methods to allow researchers to answer novel questions at increasing levels of resolution. Recent advances in molecular structure determination by cryo-electron microscopy, for example, have propelled a number of fields forward, including the provision of key insights during the development of COVID-19 vaccines (Zhang and Chen, 2022). We anticipate that new technological breakthroughs – in areas such as cryo-electron tomography, the determination of the sequences and modification states of macromolecules, and artificial intelligence – will catalyze many more important discoveries in the coming years.

NIH programs such as the Pioneer Award and the New Innovator Award, and the Maximizing Investigators’ Research Awards (MIRA) at NIGMS, provide researchers with freedom to explore new areas of science and to change direction as their work progresses. This freedom has allowed some scientists to study the biology of organisms beyond those historically used as model systems, while allowing other researchers to use emerging technologies to delve more deeply into the workings of fundamental biological processes such as DNA replication and cell division.

To accelerate the development of new technologies, several NIH institutes and centers have launched pipeline programs to provide funding for each stage of the process, from proof-of-concept to refinement and dissemination of the final product (NHGRI, 2024; NIBIB, 2024; NIGMS, 2024). As part of its support for small business development, NIH also started the Research Evaluation and Commercialization Hubs (REACH), which help small businesses in different regions of the US move new biomedical technologies to the market.

## Supporting future generations

It is also crucial that NIH maintains its longstanding commitment to training future generations of basic biomedical researchers. We must foster their interactions across a wide range of scientific disciplines and ensure that we are tapping into the immense talents that reside in every part of the US. Once these researchers have been trained, we need to redouble our efforts to fund their research as soon as possible after they start their independent positions. We need these new basic researchers to explore new scientific territory and enable the many, albeit unpredictable, breakthroughs of the future.

To enhance the retention of talented trainees in biomedical research, NIH recently launched two programs to provide support across key career transition points. The Advancing Research Careers (ARC) program provides funding for late-stage graduate students to complete their PhDs and find postdoctoral positions, and then supports them during the early stages of their postdoctoral research. The Maximizing Opportunities for Scientific and Academic Independent Careers (MOSAIC) program provides similar support for the late-stage postdoctoral to early-stage faculty transition. Both programs also fund mentoring centers that provide the scholars with career development support.

Moreover, in response to recommendations from an advisory committee, NIH recently announced significant increases in graduate and postdoctoral stipends, as well as its intention to raise the yearly postdoctoral stipend to $70,000 (NIH, 2024). In 2017, NIH launched its Next Generation Researchers Initiative to provide better support for early-career, independent researchers. This initiative set targets for NIH funding for early-stage investigators (ESIs), which led to significant increases in the number receiving their first grants – from 978 in 2016 to 1,587 in 2023 (Lauer, 2024). Through these and other programs, NIH is working to ensure a robust, diverse and vibrant workforce of well-trained basic scientists.

Although applied research might seem to be currently in the limelight, the truth is that the question should never be do we need more basic or applied research but must always be how we robustly support both. This is what NIH has done in the past and what we will continue to do in the future.

## References

[bib1] Casadevall A (2021). The mRNA vaccine revolution is the dividend from decades of basic science research. Journal of Clinical Investigation.

[bib2] Collins FS, Anderson JM, Austin CP, Battey JF, Birnbaum LS, Briggs JP, Clayton JA, Cuthbert B, Eisinger RW, Fauci AS, Gallin JI, Gibbons GH, Glass RI, Gottesman MM, Gray PA, Green ED, Greider FB, Hodes R, Hudson KL, Humphreys B, Katz SI, Koob GF, Koroshetz WJ, Lauer MS, Lorsch JR, Lowy DR, McGowan JJ, Murray DM, Nakamura R, Norris A, Perez-Stable EJ, Pettigrew RI, Riley WT, Rodgers GP, Sieving PA, Somerman MJ, Spong CY, Tabak LA, Volkow ND, Wilder EL (2016). Basic science: Bedrock of progress. Science.

[bib3] Ishino Y, Krupovic M, Forterre P (2018). History of CRISPR-Cas from encounter with a mysterious repeated sequence to genome editing technology. Journal of Bacteriology.

[bib4] Lauer MS (2024). Continued support for Early Stage Investigators in FY 2023. https://nexus.od.nih.gov/all/2024/07/03/continued-support-for-early-stage-investigators-in-fy-2023/.

[bib5] NHGRI (2024). Genome Technology Program. https://www.genome.gov/Funded-Programs-Projects/Genome-Technology-Program.

[bib6] NIBIB (2024). Division of Discovery Science and Technology. https://www.nibib.nih.gov/research-funding/division-discovery-science-technology-ddst.

[bib7] NIGMS (2024). NIGMS Technology Development Programs. https://www.nigms.nih.gov/grants/R21-R01.

[bib8] NIH (2024). NIH to increase pay levels for pre- and postdoctoral scholars at grantee institutions. https://www.nih.gov/news-events/news-releases/nih-increase-pay-levels-pre-postdoctoral-scholars-grantee-institutions.

[bib9] Petsko GA (2002). A christmas carol. Genome Biology.

[bib10] Sánchez Alvarado A (2018). To solve old problems, study new research organisms. Developmental Biology.

[bib11] Waldman AD, Fritz JM, Lenardo MJ (2020). A guide to cancer immunotherapy: Trom T cell basic science to clinical practice. Nature Reviews Immunology.

[bib12] Williams RS, Lotia S, Holloway AK, Pico AR (2015). From scientific discovery to cures: Bright stars within a galaxy. Cell.

[bib13] Wrapp D, Wang N, Corbett KS, Goldsmith JA, Hsieh CL, Abiona O, Graham BS, McLellan JS (2020). Cryo-EM structure of the 2019-nCoV spike in the prefusion conformation. Science.

[bib14] Zhang J, Chen B (2022). Fighting SARS-CoV-2 with structural biology methods. Nature Methods.

